# Tapping the biotechnological potential of insect microbial symbionts: new insecticidal porphyrins

**DOI:** 10.1186/s12866-017-1054-y

**Published:** 2017-06-27

**Authors:** Ana Flávia Canovas Martinez, Luís Gustavo de Almeida, Luiz Alberto Beraldo Moraes, Fernando Luís Cônsoli

**Affiliations:** 1 Laboratório de Interações em Insetos, Departamento de Entomologia e Acarologia, Escola Superior de Agricultura “Luiz de Queiroz”, Universidade de São Paulo, Av Pádua Dias 11, 13418–900, Piracicaba, SP Brazil; 2 Laboratório de Espectrometria de Massas Aplicada a Produtos Naturais, Departamento de Química, Faculdade de Filosofia, Ciências e Letras de Ribeirão Preto, Universidade de São Paulo, Av Bandeirantes 3900, 14040–901, Ribeirão Preto, SP Brazil

**Keywords:** *Acromyrmex coronatus*, Bacterial symbionts, Sustainable pest control, Symbiosis, Tandem mass spectrometry

## Abstract

**Background:**

The demand for sustainable agricultural practices and the limited progress toward newer and safer chemicals for use in pest control maintain the impetus for research and identification of new natural molecules. Natural molecules are preferable to synthetic organic molecules because they are biodegradable, have low toxicity, are often selective and can be applied at low concentrations. Microbes are one source of natural insecticides, and microbial insect symbionts have attracted attention as a source of new bioactive molecules because these microbes are exposed to various selection pressures in their association with insects. Analytical techniques must be used to isolate and characterize new compounds, and sensitive analytical tools such as mass spectrometry and high-resolution chromatography are required to identify the least-abundant molecules.

**Results:**

We used classical fermentation techniques combined with tandem mass spectrometry to prospect for insecticidal substances produced by the ant symbiont *Streptomyces caniferus.* Crude extracts from this bacterium showed low biological activity (less than 10% mortality) against the larval stage of the fall armyworm *Spodoptera frugiperda.* Because of the complexity of the crude extract, we used fractionation-guided bioassays to investigate if the low toxicity was related to the relative abundance of the active molecule, leading to the isolation of porphyrins as active molecules. Porphyrins are a class of photoactive molecules with a broad range of bioactivity, including insecticidal. The active fraction, containing a mixture of porphyrins, induced up to 100% larval mortality (LD_50_ = 37.7 μg.cm^−2^). Tandem mass-spectrometry analyses provided structural information for two new porphyrin structures. Data on the availability of porphyrins in 67 other crude extracts of ant ectosymbionts were also obtained with ion-monitoring experiments.

**Conclusions:**

Insect-associated bacterial symbionts are a rich source of bioactive compounds. Exploring microbial diversity through mass-spectrometry analyses is a useful approach for isolating and identifying new compounds. Our results showed high insecticidal activity of porphyrin compounds. Applications of different experiments in mass spectrometry allowed the characterization of two new porphyrins.

## Background

Despite the use of new chemistries of synthetic organic pesticides and new technologies such as genetically modified plants that express bacterial entomotoxins [1–3], the continued crop losses to insect pests necessitate the development of new tools to prevent reductions in yield and undesired effects on non-target organisms and the environment [4]. The need for integrative, sustainable management practices has led to the development of incentives and policies to support the use of integrated pest management [5], a strategy based on multiple control techniques, including the use of natural products.

The need for new compounds with insecticidal activity has increased, especially due to the evolution of insect resistance against the majority of existing insecticides and the necessity for target-specific and environmentally friendly molecules [6]. Microbes have proved to be a rich source of new bioactive molecules [7–10]. The diversity of microbes associated with insects and the selective pressures on microbes due to the range of insect habitats, have stimulated research on insect-associated microorganisms as an untapped resource for biotechnological exploitation [11–13]. Microbial symbiosis (sensu de Bary) [14] in leaf-cutting ants is well studied, including the diversity and mode of transmission of bacteria associated with the cuticle of these ants [15–17]. These ectosymbionts play a defensive role in this association by producing bioactive molecules to protect the mutualistic fungi that these ants cultivate as their food resource, from infections with parasitic fungi [18–21]. The mutualistic association of ants with bacteria has been under debate as some report *Pseudonocardia* being the mutualistic bacterium associated with the cuticle of leaf cutting ants [22, 23], while others believe the protective role of cuticle associated bacteria is provided by a diverse community [24–26]. Andersen et al. [27] provided data demonstrating *Pseudonocardia* as the prevalent bacterium growing on the laterocervical plates and pronotum of ants; but their study analysed different colonies belonging only to *Acromyrmex echinator* and a couple of other species from different genera. The prevalence of *Pseudonocardia* in the microbiota associated with the ant cuticle and their efficiency against parasitic fungi of the ant’s fungus garden certainly contributes to their role as a mutualist of ants. However, other bacterial species from the cuticle of ants were demonstrated to be more efficient in controlling the growth of the parasitic fungus *Escovopsis* than *Pseudonocardia* associated with *Acromyrmex subterraneus brunneus* [16]. *Streptomyces*-produced candicidin was demonstrated to be a powerful antibiotic against *Escovopsis* while inactive to the ant fungus garden [28]. Both studies indicate other members of the community associated with the cuticle of ants provide the same protective function as *Pseudonocardia,* supporting the proposition that the defensive contribution of the cuticle-associated microbiota is provided by a diverse community. Moreover, the ant-associated microbiota was also reported effective against entomopathogenic fungi [29], showing that these symbionts are a rich source of bioactive molecules [30] to protect the ant mutualist fungi and the ants themselves. Such controversy on the diversity of bacteria growing on the cuticle of ants and on their role in the association with ants could be a result of the varying degree of co-evolution of ants and *Pseudonocardia* [22], although phylogenetic assessment by others did not indicate any topological correspondence between *Pseudonocardia* and their host ants [31].

The identification of metabolites, a key challenge in the search for new bioactive molecules, is based on analytical techniques for structural characterization, such as mass spectrometry (MS) and nuclear magnetic resonance (NMR). Increasing the sensitivity and stability of characterization techniques has made viable studies of complex biological samples by dereplication, allowing the development of databases of metabolites to assist with the identification process [32].

Porphyrins are widely used in photodynamic therapies [33]. After photoactivation, porphyrins transfer energy to oxygen molecules that have changed their energy state (triplet to singlet), increasing oxygen reactivity and leading to cell death [34, 35]. Biotechnological applications of porphyrins are diverse [36–38] and include their successful use as insecticides [38, 39]. Porphyrins are well known for their phototoxic activity [39–42]. Photoactivity is related to the production of very toxic and highly reactive oxygen species (ROS) in the presence of UV or visible radiation. ROS is a term used for molecules and reactive intermediates with highly positive redox potentials [43]. ROS are produced when a photoactive substance, a photosensitizer (PS), is activated by low doses of UV-visible light at an appropriate wavelength. ROS can be produced as free radicals (Type I) or as singlet oxygen O_2_(_1_D_g_) (Type II). The four major ROS studied are superoxide (O_2_^**.–**^), hydrogen peroxide (H_2_O_2_), hydroxyl radical (^**.**^OH) and singlet oxygen. Singlet oxygen is formed when an electron is removed from π^*^2p orbitals of oxygen. The photoactivity observed for porphyrins is Type II [42]. Singlet oxygen is understood to play a major role in this effect, and photoactive molecules are increasingly being used in blood sterilization, cancer therapy, and insect and weed control [44].

The great advantage in the use of porphyrins compared to other photodynamic molecules is their light-absorbing capacity at all wavelengths in the UV-visible spectrum, which allows porphyrin excitation under exposure to natural light [37].

Here, we describe the successful exploration of bacteria-associated insect symbionts as sources of new bioactive molecules, and report the structures of new porphyrins with insecticidal properties against the polyphagous fall armyworm, *Spodoptera frugiperda* (J.E. Smith) (Lepidoptera, Noctuidae), based on tandem MS (neutral-loss experiment).

## Results

**Fig. 1:**
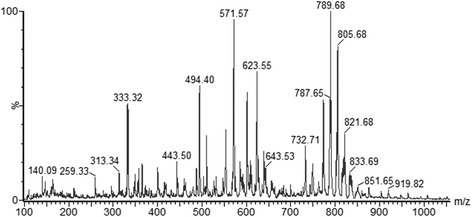
Chemical profile obtained in positive mode (ESI+) by mass spectrometry of crude extract produced by *Streptomyces caniferus*

We used a classical strategy of fermentation and isolation of bioactive compounds to prospect for molecules with insecticidal activity produced by ectosymbionts of the leaf-cutting ant *Acromyrmex coronatus*. Our preliminary screening identified an isolate (IIL-Ac-18dV) that was tentatively identified as *Streptomyces caniferus* because it shared 99.98% identity over 1350 bp of 16S rRNA with the type strain (AB184640). This isolate produced a complex crude extract (Fig. 1) with low insecticidal activity (approximately 10% mortality) against first instars of *S. frugiperda*.

Preliminary experiments by Collision-induced dissociation (CID) of the crude extract of this isolate led to the identification of three chemical classes of compounds in the regions at *m/z* 540–650, *m/z* 730–780 and *m/z* 785–840.

Our experience with isolation of bioactive compounds produced by microorganisms indicates that these compounds are present in low concentrations in most cases. Therefore, we believed that biological activity could not be detected due to the very low concentration of the active compound in initial screenings. To prove this supposition, bioactivity and isolation experiments were conducted with the crude extract, which showed only 10% mortality in the initial screening, and these experiments led to the isolation and identification of an active compound. The higher activity after fractionation and isolation confirmed the complexity of the original crude extract and indicated that the lack of observation of porphyrin signals in the mass spectrum of the original crude extract was likely due to ionization suppression.

Toxicity-driven fractionation of the crude extract from the isolate IIL-Ac-ASP18v allowed the isolation of a fraction that contained a mixture of three porphyrins. Bioassays of this fraction against third instars of *S. frugiperda* indicated high insecticidal activity, with a LD_50_ of 37.7 μg.cm^−2^ (*y* = 1.876–1.051*×*; *n* = 72; *df* = 3; *χ* ^2^ = 6.0723; *P* < 0.05). Larvae exposed to the porphyrin fraction turned black, which could be related to the oxidative process in cells and tissues exposed to porphyrins.

The purified porphyrins *coproporphyrin* and *zinc coproporphyrin III* using the estimated LD_50_ concentration caused approximately 30% mortality in third instars of *S. frugiperda*.

### Isolation and characterization of porphyrins in the active fraction

**Fig. 2:**
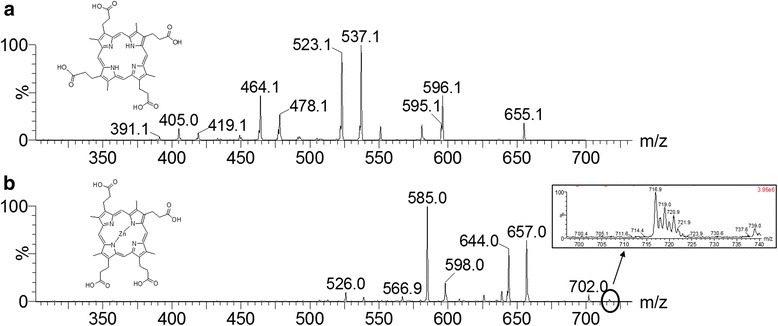
Structure and CID spectrum of **a**) *coproporphyrin I* (*m/z* 655) and **b**) *zinc coproporphyrin III* (*m/z* 717). The insert in **b** illustrates the isotopic pattern of zinc in the molecule

Porphyrin signals were observed at *m/z* 655, 669 and 717. The CID of an ion at *m/z* 655 produced a fragmentation profile that allowed the characterization of *coproporphyrin I*, as corroborated by comparison with data in the literature [45, 46]. The CID spectrum of *m/z* 655 produced a neutral loss of 74 Da (*m/z* 581), which corresponded to losses of propionic acid (CH_3_CH_2_COOH). α-cleavage was observed in losses of 59 Da. Four consecutive losses of 59 Da were observed at *m/z* 596, 537, 478 and 419. Losses of 59 Da revealed radical losses. Consecutive losses of 59 and 73 Da were also observed at *m/z* 596 [M-59 + H]^+^, *m/z* 523 [M-59-73 + H]^+^, *m/z* 464 [M-59-73-59 + H]^+^ and *m/z* 391 [M-59-73-59-73 + H]^+^. Base peak *m/z* 537 corresponds to a loss of 118 Da, which corresponded to one radical of propionic acid (^.^CH_2_CH_2_COOH) and three methyl groups (^.^CH_3_), while a loss of 132 Da (*m/z* 523) corresponded to losses of four methyl groups and one propionic acid residue. All losses followed radical mechanisms. The CID spectrum at *m/z* 655 and the predicted structure of this molecule are illustrated in Fig. 2a. The theoretical value of log *P* obtained for *coproporphyrin I* was 5.22.

One loss of 60 Da (*m/z* 657) and three consecutive losses of 59 Da (*m/z* 598, 539 and 480) were observed for an ion at *m/z* 717. The fragmentation profile of the porphyrin at *m/z* 717 differed in showing losses of 72 Da for ions at *m/z* 585 [M-60-72 + H]^+^ or *m/z* 526 [M-60-59-72 + H]^+^ or *m/z* 467 [M-60-59-59-72 + H]^+^. The ion at *m/z* 644 corresponded to a loss of 73 Da (^.^CH_2_CH_2_COOH). This structure corresponded to *zinc coproporphyrin III*. The CID spectra, isotopic profile and structure of *zinc coproporphyrin III* are illustrated in Fig. 2b.

**Fig. 3:**
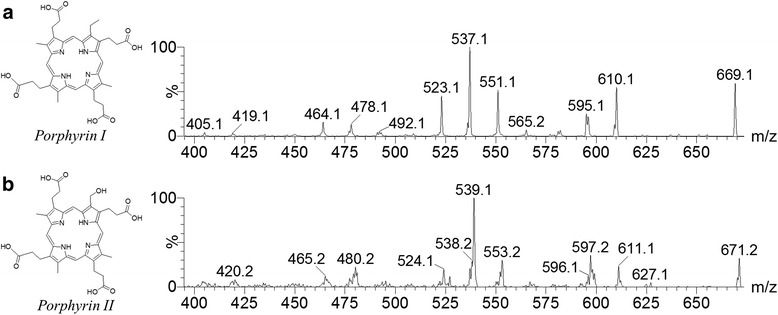
Structures and CID spectra of new insect-associated microbial porphyrins. **a**) *porphyrin I* (*m/z* 669); **b**) *porphyrin II* (*m/z* 671)

Another porphyrin (*porphyrin I*) was available in the active fraction at *m/z* 669. The same fragmentation profile observed for an ion at *m/z* 655 was observed for this porphyrin. The fragmentation profile obtained for *m/z* 669 by CID analysis produced four consecutive α-cleavages, observed in radical losses of 59 Da (*m/z* 610, 551, 492 and 433) and neutral loss of 74 Da (*m/z* 595). Consecutive losses of 59 and 73 Da were also observed: *m/z* 610 [M-59 + H]^+^, *m/z* 537 [M-59-73 + H]^+^, *m/z* 478 [M-59-73-59 + H]^+^ and *m/z* 405 [M-59-73-59-73 + H]^+^. The signal at *m/z* 523 corresponded to loss of 146 Da, i.e. loss of one propionic acid residue, three methyl radicals (^.^CH_3_) and one ethyl radical (^.^CH_2_CH_3_). The theoretical log *P* value for hydrophobicity of *porphyrin I* was 5.64. The CID spectrum at *m/z* 669 and the predicted structure of this molecule are illustrated in Fig. 3a.

### Neutral-loss experiments

**Fig. 4:**
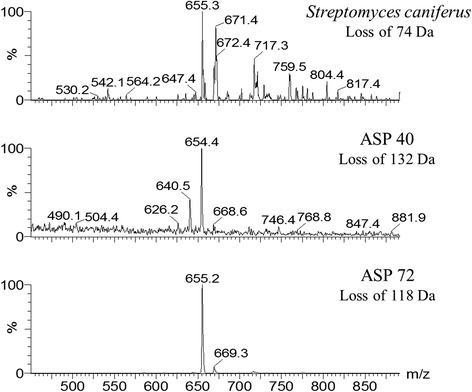
Spectra of neutral-loss experiments for *Streptomyces caniferus, Streptomyces aureus* and *Streptomyces eurocidicus*

Neutral-loss experiments for monitoring the availability of porphyrins in 67 crude extracts of insect microbial symbionts led to the identification of porphyrins in the extracts produced by isolate IIL-Ac-ASP40, putatively identified as *Streptomyces olivochromogenes* (99% identity over a 1350-bp fragment of the 16S rRNA gene) and IIL-Ac-ASP72, putatively identified as *Streptomyces eurocidicus* (98.96% identity over a 1350-bp fragment of the 16S rRNA gene) (unpublished data). Porphyrins were identified by monitoring signals with 118 Da, 132 Da, 74 Da and 60 Da, all products of fragmentation of *coproporphyrin I* (Fig. 2a). Isolates IIL-Ac-ASP40 and IIL-Ac-ASP72 produced two of the same porphyrins produced by isolate IIL-Ac-ASP18v, *coproporphyrin I* at *m/z* 655 and the new *porphyrin I* at *m/z* 669 (Fig. 3a). Neutral-loss experiments on the crude extract of isolate IIL-Ac-ASP18v resulted in the discovery of a new ion at *m/z* 671, allowing the identification of a fourth porphyrin (*porphyrin II*) (Figs. 3b and 4).

The CID experiment resulted in an interesting fragmentation profile for ion *m/z* 671. This structure showed the most distinct fragmentation profile, with neutral losses predominating over radical losses. α-cleavages were observed, with losses of 60 (neutral loss) or 59 (radical loss) Da. The signal at *m/z* 522 corresponds to the loss of 148 Da, i.e. loss of one propionic acid residue, three methyl radicals (^.^CH_3_) and one methanol radical (^.^CH_2_OH). The theoretical log *P* value for hydrophobicity of *porphyrin II* was 4.1. The CID spectrum at *m/z* 671 and the predicted structure of this molecule are illustrated in Fig. 3b.

## Discussion

We demonstrated the potential of insect microbial symbionts to produce new molecules with insecticidal activity against the lepidopteran *S. frugiperda*, describing three new structures of photoactivated porphyrins. We also demonstrated that detailed structure analysis and molecule bioassay-guided isolation of active compounds from complex crude extracts with low biological activity can result in the isolation of new, highly active molecules: the new *porphyrins I* and *II*. The porphyrins that were characterized in this study would not have been identified, nor would their insecticidal potential have been detected if we had followed a traditional approach, as the low proportion of porphyrins in the crude extract did not produce a promising insecticidal activity in the initial screening. Our results indicated that porphyrins can be used for insect control at low concentrations, as we demonstrated using third instars of *Spodoptera frugiperda*.

The insecticidal activity of the purified porphyrins *coproporphyrin* and *zinc coproporphyrin III* was lower than expected from the calculated LD_50_. This lower than expected mortality could be related to synergism among the different porphyrins in the whole fraction, and/or the additional manipulation for porphyrin purification having allowed some degradation. For example, the antineoplastic activity and in vitro photophysical properties of Photofrin photodynamic therapy, also based on a mixture of porphyrins, are uncertain, owing to the variable pharmacokinetics and photochemistry of the constituents [47].

We also demonstrated the importance of tandem mass-spectrometry experiments for the discovery of new compounds. Neutral-loss experiments allowed the identification of *porphyrin II* (*m/z* 671) in a crude extract, which had not been identified in fractions enriched with porphyrins.

Isolate IIL-Ac-ASP18v produces porphyrins with high structural diversity and hydrophobicity. *Porphyrin II* is highly hydrophilic (log *P* 4.16), while *porphyrin I* (log *P* 5.64) shows a similar hydrophobicity to *coproporhyrin I* (log *P* 5.22). The phototoxicity of porphyrins seems to depend on their structure, as their phototoxic activity increases with their hydrophobicity [47]. Therefore, the use of these compounds in photodynamic therapy must rely on the capacity of photosensitizers to move across the cell membrane, which will depend on their hydrophobicity. Molecules with log *P* values between 8 and 10 are considered moderately or highly hydrophobic, and are likely to be the most toxic as they can diffuse through cell membranes, move into the cell and become integrated into subcellular membranes such as the mitochondrial and lysosomal membranes, Golgi apparatus and rough endoplasmic reticulum. However, even less-hydrophobic compounds can actively or passively diffuse through the cell membrane if electrically charged, due to ionic interactions with groups at the cell surface. Cationic compounds are often located in mitochondria, while anionic compounds are accumulated at the lysosomal level [39].

Ben-Dror et al. [48] employed a series of synthetic porphyrins (log *P* between 7.4 and 10.5, as estimated with the ChemDraw software) with peripheral modifications that were expected to change the hydrophobicity with no change to the chromophore group. They measured the binding constant to liposomes, and determined that in highly hydrophobic porphyrins, neither the lipophilicity nor the passive binding to liposomes adequately predicted the cell uptake. In these cases, log *P* is not the only factor that should be used to predict porphyrin activity, and should be evaluated with caution as this may produce misleading conclusions, as in the case of passive uptake into liposomes, or be irrelevant in cases where other uptake mechanisms predominate, as with cells and highly apolar molecules. The singlet-oxygen production showed no significant changes.

Photosensitizers such as porphyrins generally lack cytotoxicity in the absence of light, which could be an advantage in biological systems where rapid breakdown of the photosensitizer after use is necessary. However, for industrial applications this is an undesirable aspect [44].

Similarly to other photosensitizers (e.g. xanthenes), porphyrins have been reported to affect the epithelial membrane of the midgut of insects and to inhibit insect feeding [39]. Amor et al. [41] discussed the efficiency of hematoporphyrin against *Ceratitis capitata* (Mediterranean fruit fly), *Bactrocera* (*Dacus*) *oleae* (olive fruit fly) and *Stomoxys calcitrans* (stable fly). *Ceratitis capitata* showed about 70% mortality after 1 h exposure to 11–12 μmol.mL^−1^ (approximately 6.5 mg.mL^−1^) of hematoporphyrin; while in our experiment, the active fraction of porphyrins produced by IIL-Ac-18 V showed an LD_50_ of 3.6 mg.mL^−1^ (37.7 μg.cm^−2^). *Bactrocera* (*D.*) *oleae* showed lower photosensitivity than did *C. capitata.* Additional data on the efficacy of porphyrin mixtures for *C. capitata* control also indicated the importance of the porphyrin structure to their insecticidal activity. The amphiphilic cationic porphyrin DDP (log *P* 20) produced high mortality, while hematoporphyrin (log *P* 12) was nearly atoxic [48]. However, high hydrophobicity can also lead to porphyrin aggregation, which would reduce its photoactivity [47]. These results reveal the importance of the porphyrin structure, and log *P* does not necessarily correlate directly with porphyrin biological activity.

Before field application can be recommended, important aspects of porphyrin-plant interactions must be considered based on experience with the use of tetrapyrrole-dependent photodynamic herbicides (TDPH). TDPH have been considered phytotoxic to certain plants, which are sprayed in the dark a few hours before they are exposed to light. Once exposed to light, the plants can accumulate massive amounts of tetrapyrroles, and damage appears after 20 min, becoming irreversible after 60 min. The toxicity of TDPH to plants is age- and species-dependent: dicotyledonous weeds such as mustard, red-root pigweed, common purslane and lamb’s quarter are very susceptible, while monocotyledonous plants such as corn, wheat, barley and oats are not [49, 50]. Therefore, the use of new phorphyrins as described here to control chewing insects such as the fall armyworm in field applications will require additional assays to check for plant toxicity and photodegradation that may occur once the molecules are exposed to light.

## Conclusions

Our results demonstrated the importance of bacterial insect symbionts as sources of new and/or bioactive compounds. We used an unusual approach to pursue the isolation of bioactive molecules, by focusing on crude extracts with low insecticidal activity, aiming to identify bioactivity among the least-abundant molecules in the crude extract. The crude extracts from IIL-Ac-ASP18v showed a complex chemical composition after analysis by direct insertion in the mass spectrometer, in positive mode (ESI+). After isolation of the porphyrin fraction, the insecticidal activity induced up to 100% larval mortality (LD_50_ = 37.7 μg.cm^−2^), proving our strategy successful. The use of sensitive analytical tools such as mass spectrometry allowed the characterization of two new porphyrins.

## Methods

### Microorganisms: Isolation and identification

Microorganisms were isolated from the leaf-cutting ant *Acromyrmex coronatus* (Hymenoptera, Formicidae) using ISP2 [51], ISP4 [51] and chitin [52] as culture media. The isolates obtained were identified based on 16S rRNA analysis (unpublished data). These isolates were then used to screen for insecticidal activity and porphyrin biosynthesis.

### 16S rRNA gene sequencing analysis

A selected bacterial isolate (IIL-Ac-18dV) was grown at 28 °C for 24 h under constant agitation (120 rpm) in 1 mL of the same culture medium used in the initial isolation. Cells were precipitated by centrifugation (2000 *g* × 5 min) and then used for genomic DNA extraction [53]. DNA quality and integrity were assessed after electrophoresis in 0.8% (*w*/*v*) agarose gel containing 0.5 μg/mL ethidium bromide at 70 V for 1 h in TAE buffer (40 mM Tris-acetate, 1 mM EDTA, pH 7.2), and spectrophotometry analysis using the A260/280 ratio [54, 55]. DNA samples were subjected to PCR amplification of a fragment of the 16S rRNA gene using 10–20 ng of genomic DNA and the universal primer set 8f (5′-AGA GTT TGA TCC TGG CTC AG-3′) and 1491r (5′-GGT TAC CTT GTT ACG ACT T-3′) [56] in 1× enzyme buffer, 1.5 mM MgCl_2_, 0.2 mM dNTPs, 0.32 μM of each primer, and 0.625 U of Taq polymerase (Promega), in a final reaction volume of 25 μL. PCR cycling conditions were 4 min at 95 °C (1×); 95 °C for 1 min, 55 °C for 1 min, and 72 °C for 2 min (35×), followed by a final extension at 72 °C for 10 min (1×). The 16S rRNA fragment obtained was submitted to bidirectional sequencing at the Laboratório de Biologia Molecular de Plantas, Departamento de Ciências Biológicas, ESALQ/USP, in an ABI 3730 DNA Analyzer using the BigDye® Terminator v3.1 Cycle Sequencing kits. The sequence obtained was viewed and edited using Finch TV v1.4.0 (Geospiza Inc.) and forward and reverse reads were assembled in a single ~1350 bp-long read using the Blast2 tool. The 16S rDNA fragment obtained was used in heuristic blast searches against the nucleotide databases of the National Center for Biotechnology Information (NCBI) (http://www.ncbi.nlm.nih.gov/) and EzTaxon [57] and (http://www.ezbiocloud.net/) for the tentative identification of the isolated bacterium. The sequence obtained for the isolate was deposited in GenBank under accession number KX762323.

### Crude extract preparation

The selected isolate (IIL-Ac-18dV) and other microorganisms from our bank of isolates were inoculated in ISP2 medium and cultured for 7 d at 28 °C under constant shaking (130 rpm). Crude extracts were obtained by liquid-liquid extraction with one volume of ethyl acetate (Synth), following standard procedures [58].

### Isolation of porphyrins for insecticidal bioassay

Isolate IIL-Ac-18dV was inoculated in 4.5 L of ISP2 medium. After liquid-liquid extraction, 180 mg of crude extract was produced. The crude extract was purified using a Sephadex™ LH-20 (particle size range: 27–163 μm; mean diameter: 103 μm; GE Healthcare, Sweden) - packed column (300 × 20 mm), with methanol (J.T. Baker) as the eluent. A total of 29 fractions, including an initial fraction (30 mL) + 27 fractions (12 mL per fraction) + a final fraction (50 mL), were collected. Eluted compounds were monitored for color and UV absorbance (λ = 254 and 356 nm). Pink fractions indicative of porphyrins were separated and analyzed by MS/MS experiments and subjected to biological assays.

### Chromatographic conditions for porphyrin purification

Chromatographic analyses were performed in an Ultra High-Performance Liquid Chromatography system (UHPLC Accela 600, Thermo Scientific) equipped with a diode array detector, auto sampler and an ACE 5 column (250 × 4.6 mm; 5 μm). Porphyrin was monitored at λ = 400, 533 and 575 nm. Samples were eluted using a gradient of 0.1% formic acid (phase A) and methanol/0.1% formic acid (phase B). The gradient started with 90% phase B and increased linearly to 95% within 3 min, with an additional hold for 7 min in 95% phase B. The flow rate was 1 mL.min^−1^. Peaks were purified using an ACE 5 column (250 × 7.75 mm, 5 μm) under the same elution gradient at a flow rate of 2.8 mL.min^−1^.

### Bioassay

#### Screening

The insecticidal activity of isolate IIL-Ac-18dV was evaluated against first instars of the fall armyworm *Spodoptera frugiperda* (Lepidoptera, Noctuidae) by exposing the larvae to a surface-treated artificial diet. The artificial diet used was that of Kasten et al. [59], and insect rearing and handling followed Parra [60]. Bioassays were conducted in sterile 24-well plates filled with 1.25 mL of artificial diet. After the diet solidified, 20 μL of the test solution was applied to the diet surface. In the initial screenings of crude extracts, samples were diluted in methanol to 25 μg.μL^−1^, resulting in the application of 500 μg of crude extract in each cell (260 μg.cm^−2^). Methanol was used as the negative control. After the solvent dried completely, each cell was inoculated with ten (10) 24 h-old larvae, totaling 240 larvae/treatment. Plates were maintained under controlled conditions (25 ± 2 °C; 60 ± 10% RH; 14 h photophase), and larval development and mortality were evaluated daily for 3 d. Mortality was assessed by touching the last abdominal segments, and larvae that were unresponsive or showed uncoordinated movements were considered dead.

#### Porphyrin bioassay

Insecticidal activity of the purified porphyrin fraction produced by isolate IIL-Ac-18dV was evaluated using third instars of *S. frugiperda,* at 1, 2, 8, 16, and 65 μg.cm^−2^. Bioassays were performed as above, but only three third instars were placed in each well of the plates, totaling 72 larvae/treatment. Plates were maintained under the same conditions, and larval development and mortality assessed as above. The program PoloPlus 1.0 was used to determine the dose-response curve and LD_50_ concentration, by Probit analysis (Leora Software, 1987).

The two major porphyrins (*coproporphyrin* and *porphyrin II*) in the porphyrin fraction were isolated and individually tested for insecticidal activity against third instars of *S. frugiperda* at the estimated LD_50_ concentration for the whole porphyrin fraction, as described above.

#### MS and MS/MS experiments

The crude extract and porphyrin-enriched fractions were analyzed by direct insertion in a Xevo™ TQ-S (Waters Corporation) mass spectrometer coupled with Acquity™ Ultra High-Performance Liquid Chromatography (UPLC™, Waters). The mass spectrometer operated with electrospray ionization in the positive mode (ESI+). The flow rate was 0.15 mL.min^−1^. The capillary voltage and the spray voltage were set to 3.3 kV and 60 V, respectively; the desolvation temperature was 350 °C; and argon was used as the collision gas.

#### Porphyrin characterization

Fractions obtained from the crude extract were analyzed by direct insertion in a mass spectrometer operating in positive (ESI+) and negative (ESI–) modes, in a full scan experiment, and fractions with similar chemical compositions were pooled. The porphyrins present in the active fraction were characterized with collision-induced dissociation (CID) experiments employing *coproporphyrin* I, due to the availability of the fragmentation profile in the literature [45, 46]. Several collision energies were tested (20–70 V) before 50 V was selected as the collision energy. The concentration of the porphyrin fractions was 5 μg.mL^−1^ in methanol. The hydrophobicity of the porphyrins identified was also estimated, by determining the partition coefficient between an organic solvent and water, or log *P*, a measurement normally used to predict the ability of a molecule to diffuse into biomembranes. Theoretical values of log *P* for the porphyrins produced by isolate IIL-Ac-ASP18v were calculated using the commercial software Chem DrawPro 8.0 (CambridgeSoft Corporation).

#### Neutral-loss experiment

Neutral-loss experiments were performed in order to search for porphyrins in crude extracts from 67 ant-ectosymbiont isolates (crude extract preparations were obtained as described above). Selected neutral losses were 60 Da, 74 Da, 118 Da and 132 Da. These neutral losses were selected based on the main fragments found in CID experiments for *coproporphyrin I*, the major porphyrin present in the active fraction of isolate IIL-Ac-18dV and previously described [45, 46], which allowed the dereplication studies for this compound. The collision energy employed was 50 V. The concentration of the crude extracts was 5 μg.mL^−1^ in methanol. Ions that showed two losses were analyzed by daughter scan experiments to obtain structural information.

#### LC-MS conditions

The analyses of isomers of porphyrins and of the crude extracts obtained under acidic conditions were conducted on a LC-MS in a Xevo TQ-S (Waters Corporation) Mass Spectrometer coupled with Acquity Ultra High-Performance Liquid Chromatography (UPLC, Waters). Samples were eluted using a gradient of 0.1% formic acid (phase A) and methanol/0.1% formic acid (phase B). The gradient started with 40% phase B and increased linearly to 95% within 5 min, with an additional hold for 2 min in 95% phase B. The flow rate was 0.4 mL.min^−1^. The mass spectrometer operated with electrospray ionization in the positive mode (ESI+). The capillary voltage and spray voltage were set at 3.0 kV and 40 V, respectively; with desolvation temperature 300 °C, source temperature 120 °C, argon used as the collision gas, and collision gas flow 0.2 mL.min^−1^.

## Abbreviations

CID: Collision-induced dissociation
Da: Daltons
DPP: diketopyrrolopyrrole
LD: lethal dose
MS: mass spectrometry
PS: photosensitizer
ROS: reactive oxygen species
rRNA: ribosomal ribonucleic acid
UV: ultraviolet
